# The clinical learning environment during clinical practice in postgraduate district nursing students' education: A cross‐sectional study

**DOI:** 10.1002/nop2.1356

**Published:** 2022-09-05

**Authors:** Margaretha Larsson, Annelie J. Sundler, Karin Blomberg, Birgitta Bisholt

**Affiliations:** ^1^ Institution of Health Sciences University of Skövde Skövde Sweden; ^2^ Faculty of Caring Science, Work Life and Social Welfare University of Borås Borås Sweden; ^3^ Faculty of Medicine and Health, School of Health Sciences Örebro University Örebro Sweden; ^4^ Institution of Health Sciences Red Cross University Stockholm Sweden

**Keywords:** clinical practice, community‐based health care, learning, postgraduate nursing education, preceptor, primary health care, second‐cycle education, specialist nurse education, supervision

## Abstract

**Aim:**

To describe and compare the clinical learning environment in community‐based home care and primary health care in postgraduate district nursing students' education.

**Design:**

Cross‐sectional study design.

**Methods:**

A convenience sample of postgraduate district nursing students was derived from five Swedish universities in 2016 and 2017.

**Results:**

The postgraduate district nursing students were generally satisfied with the clinical learning environment in their clinical placement. In clinical placement, several factors affected the students' opportunities to learn, such as sufficiently meaningful learning situations with multidimensional content. A working environment that imposed psychosocial strain and high levels of stress among the staff negatively affected the students' learning. To further improve their learning from clinical practices, the students need preceptors who have the skills and competence required to support more advanced reflections and critical thinking on caring situations.

## 1 INTRODUCTION

The clinical learning environment is important for quality nurse education (Rodríguez‐García et al., 2021; Saukkoriipi et al., 2020; Sundler et al., 2014) and postgraduate nurse education (Ginger & Ritchie, 2017; Sundler et al., 2019). Research has indicated that supervision during clinical practice, in terms of discussions and reflections, is important for postgraduate nursing students' learning experiences and satisfaction (Sundler et al., 2019). However, to our knowledge, few studies focuses on postgraduate nurses' experience of the clinical learning environments in community‐based home care and primary health care (Jarnulf et al., 2019; Sundler et al., 2019). More knowledge is needed to develop clinical practice and postgraduate district nursing education. This study focused on describing and comparing clinical learning environments in community‐based home care and primary health care in postgraduate district nursing students' education.

## 2 BACKGROUND

Primary health care and community‐based home care are two rapidly growing central areas of nursing, relating to health promotion (Irvine, 2005), the prevention and management of long‐term illness and palliative care (WHO, 2021). Due to an ageing population, increasing demands are being placed on healthcare systems to improve the health of citizens (WHO, 2018). This is in turn placing an increased demand on healthcare systems to have a sufficient number of healthcare practitioners with appropriate skills and competences.

Dury et al. (2014) noted that specialist nursing education in Europe is unclear and that there are differences in the required level and duration of specialist nursing education across European countries. Since 2007, as a result of European education reform, specialist nurse education in Sweden, similar to many other countries, has second‐cycle higher education of 60 to 75 credits in the European Credit Transfer System, including a master's degree upon graduation as a nurse with a bachelor's degree. Specialist education develops student nurses' skills in critical and ethical thinking and to work evidence‐based (Wijk et al., 2009). Millberg et al. (2014) argued that the academic skills acquired during specialist nurse education were not just valuable but necessary for students to meet the professional demands placed upon them.

In Sweden, district nursing is the most common type of specialist nurse education (Socialstyrelsen & Universitets‐kanslers‐ämbetet, 2019) and is offered at the postgraduate level by 17 universities, with nearly 540 enrollees every year (SCB, 2021). However, due to an expanding population, particularly an increased proportion of children, adolescents and elders (SCB, 2021)—circumstances also prevalent in the rest of the world (United Nations, 2021)—there is an increasing shortage of district nurses. To recruit more district nursing students, universities should provide interesting and relevant education of good quality throughout the whole specialist district nurse programme. One of the main challenges in this regard is creating meaningful clinical placements that allow students to develop their skills based on new knowledge and general nursing competences (Cretu & Stilos, 2021; Yiend et al., 2016). Clinical education is considered a key feature in all nursing programmes since it enables students to connect their theoretical knowledge to practice in actual clinical events (Phillips et al., 2017). In nursing education, Flott and Linden (2016) described four components of the clinical learning environment: the physical space, psychosocial and interaction factors, organizational culture and teaching and learning. A good clinical learning environment consists of a climate characterized by effective communication between students and nurse preceptors (Phillips et al., 2017) and varied learning situations, both of which can affect the professional development of the students (Jans et al., 2021). A review of the literature on clinical learning environments at the postgraduate level revealed that few studies have highlighted the views of district nursing students. Sundler et al. (2019) reported that district nursing students valued to have several preceptors, who provided a broader picture of and different views about working as a specialist nurse and preceptors who acknowledged the students' previous work experiences. Ginger and Ritchie (2017) claimed that evaluations between preceptors and district nursing students during clinical practice can encourage the students to identify and discuss their ongoing learning needs and directions for further learning.

Although primary health care and community‐based home care are essential aspects of the clinical learning environment for postgraduate district nursing students, more knowledge of their learning in clinical practice is needed. Increased understanding and knowledge about important aspects of the clinical learning environment can contribute to optimizing students' learning and ensuring patient safety. Therefore, this study aims to describe and compare clinical learning environments in community‐based home care and primary health care in postgraduate district nursing students' education.

## 3 METHODS

### 3.1 Design

A cross‐section design was used to explore the clinical learning environment as rated by specialist nurse students. The study is a part of a research program (L‐Pro). A questionnaire survey was used to collect quantitative and qualitative data. The questionnaire was administered to students enrolled in Specialist Nurse Education for District Nursing at five Swedish universities (henceforth district nursing students).

### 3.2 Sample and setting

In this study, district nursing students from Karlstad University, Örebro University, University of Skövde, University of Mälardalen and University Borås were asked to describe their views of their clinical practice at placements in primary healthcare centres and community‐based home care over a period of between three and 5 weeks. All students were supervised in accordance with the respective course syllabus. The goal is for preceptors to have a specialist nurse degree, although this was not always possible. Basic eligibility for admission to any specialist nursing programme requires a Registered Nursing degree and a bachelor's degree. In order to take a specialist district nurse exam, the students must fulfil course requirements of 75 higher education credits (i.e. 50 weeks of studies), which are regulated in part by the national Higher Education Ordinance (SFS:, 1993:100) and in part by the curriculum of each university.

### 3.3 Data collection

Data were collected in 2016 and 2017 by means of a questionnaire administered to postgraduate district nursing students approximately 1 week after they had finished their clinical practice. The questionnaire was distributed by the researchers with the assistance of teachers. The first part of the questionnaire contained questions about gender, age, duration of work as a Registered Nurse and study rate. The second part of the questionnaire included questions about clinical practice, that is the length of clinical practice, the number of students in the clinical setting, the type of supervision and whether the student had met with a clinical teacher from the university. There was also a question about total satisfaction with clinical practice with response alternatives measured on a 5‐point Likert‐type scale. Higher values indicated greater satisfaction. The third part of the questionnaire contained an instrument, the Clinical Learning Environment, Supervision and Nurse Teacher (CLES+T) scale, to evaluate the clinical learning environment as perceived by the district nursing students. The last part of the questionnaire consisted of four open‐ended questions: “Was the timing of this clinical practice of importance to you? Can you describe facilitators for your learning environment during this clinical practice? Can you describe barriers for your learning environment during clinical practice? Do you have anything additional to add?” In this study, the data about facilitators of and barriers to learning in clinical environments were used.

### 3.4 The clinical learning environment, supervision and nurse teacher (CLES+T) scale

The CLES+T scale consists of 34 statements divided into five sub‐dimensions: “Pedagogical atmosphere on the ward” (nine items), “Leadership style of the ward manager” (four items), “Premises of nursing on the ward” (four items), “Supervisory relationship” (eight items) and “Role of the nurse teacher (NT)” (nine items). Responses to the CLES+T items are measured on a 5‐point Likert‐type scale, with higher values indicating greater agreement with the statement. The response options are (1) “fully disagree”, (2) “disagree to some extent”, (3) “neither agree nor disagree”, (4) ‘agree to some extent’ and (5) “fully agree” (Saarikoski et al., 2008; Saarikoski & Leino‐Kilpi, 2002).

Evidence for the validity of the original scale based on its content has been generated by review of the empirical literature (Saarikoski et al., 2008). In this study, we used the Swedish version of the CLES+T, which has proven to be a reliable and valid instrument in psychometric tests among Swedish student nurses (Gustafsson et al., 2015; Johansson et al., 2010). Ozga et al. (2020) found the psychometric properties of the CLES+T to be satisfactory and considered it to be a useful instrument for assessing aspects of clinical learning environments that are important for ensuring the quality of education at the postgraduate level. Statistical analyses of Cronbach's coefficient alpha demonstrated that the internal consistency of the scale as a whole and its five sub‐dimensions was good to excellent. The alpha value was .95 for the total scale and ranged from .77 to .95 for its sub‐dimensions.

### 3.5 Statistical analyses

Students who had completed placements in primary healthcare centres and community‐based home care were divided into two independent groups. Statistical analyses of differences between the students about their clinical placements were performed by means of a chi‐squared test for data on nominal scales and a Mann–Whitney U‐test for data on ordinal scales. The CLES+T instrument obtains data on the ordinal‐scale level. Responses to the statements for each sub‐dimension of the CLES+T were described with mean values (m) and standard deviations (*SD*) to enable comparisons with previous studies. When single responses were missing, they were replaced with a score that did not change the students' individual mean value for the sub‐dimension. Differences between the groups were analysed with a Mann–Whitney U‐test. This test is non‐parametric and takes into account that the responses are given on ordinal scales. We used IBM SPSS version 24 for the statistical analyses. The significance level was set to .05.

### 3.6 Qualitative content analysis

The data from the open‐ended questions were analysed according to directed content analysis as described by Hsieh and Shannon (2005).

### 3.7 Ethical considerations

The study was conducted according to the Code of Ethics of the Declaration of Helsinki (WMA Declaration of Helsinki, 2013) and the ethical guidelines for nursing research in Swedish law (SFS, 1993:100). The Regional Ethical Review Board in Uppsala (reg. no. 2011/071) determined that no Research Ethics Committee approval was needed for the study according to Swedish law since it did not fall in the scope of the Act (SFS, 2003:460) on ethics testing of human‐related research.

The participants received verbal and written information about the study after they had completed their clinical practice and had returned to the university campus. They were informed that their participation was entirely voluntary and that they could withdraw from the study at any time until the analysis had begun without any consequences for their education. Before data collection began at each university, informed, written consent was obtained from all participants. All data were collected anonymously on paper in sealed envelopes submitted to a teacher. The researchers were not involved in assessments or examinations of the participants at the time of participant recruitment and data collection.

## 4 RESULTS

### 4.1 Participant characteristics

The study sample consisted of 128 postgraduate district nursing students who ranged in age from 26 to 55 (mean 38 years). Most of the students were female (*n* = 123, 96%), which corresponds to the ratio of female to male students in Swedish postgraduate district nursing education. Prior to beginning their studies, the students had worked as Registered Nurses for 2–29 years (median 9 years). The students had the opportunity to choose their study rate, with 113 students (88%) choosing full‐time study and 15 students choosing part‐time study. The latter worked as nurses in parallel with their studies. All clinical placements were full‐time, as only university courses were possible to fulfil part‐time.

### 4.2 Clinical placement

About one‐half of the district nursing students had performed their clinical placement in primary health care (*n* = 66, 52%), with the remainder having performed their clinical placement in community‐based home care (*n* = 62, 48%). The length of their clinical placement varied between 3–5 weeks and differed statistically significantly between primary health care and community‐based home care settings (Table 1). Placements in community‐based home care never exceeded 4 weeks, whereas placements in primary health care lasted 5 weeks at most. In general, each respondent was the only student in the clinical setting, although as many as 10 students could be performing their placement at the same time in community‐based home care and five students could be performing their placement at the same time in primary health care. When asked about their experience of supervision, 50 students (39%) reported being supervised by a personal preceptor, 50 (39%) by several preceptors, and 28 (22%) stated that they mainly worked independently with no or very little supervision. The type of supervision differed statistically significantly between clinical placements. In community‐based home care, supervision by a personal preceptor was the most frequent type; while in primary health care, supervision by several preceptors was the most frequent type. It was also more common for students to work independently in primary health care. Of the district nursing students, 91 (71%) had met with a nurse teacher from the university during their clinical placement. Those students performing their placement in primary health care had met with a nurse teacher from the university more often than had students in community‐based home care. The evaluation of total satisfaction with the clinical placement showed that, in general, the students were satisfied with their placement. However, those students who had performed their clinical placement in community‐based home care were statistically significantly less satisfied compared to those who had performed their clinical placement in primary health care (Table 1).

**TABLE 1:** Differences between student nurses with regard to clinical practice in community‐based home care (*n* = 66) and primary healthcare centres (*n* = 62)

	Total (*n* = 128)	Community‐based Home care (*n* = 62)	Primary healthcare centres (*n* = 66)	*p*‐value
Length of clinical practice^a^, *n* (%)				
3 weeks	29 (23%)	18 (29%)	11 (17%)	<.001^b^
4 weeks	73 (57%)	44 (71%)	29 (44%)	
5 weeks	26 (20%)	0	26 (39%)	
Number of students in the clinical setting, md (range)	1 (0–10)	1 (0–10)	1 (0–5)	.200^c^
Type of supervision, *n* (%)				
Supervised by a personal preceptor	50 (39%)	38 (61%)	12 (18%)	<.001^b^
Supervised by several preceptors	50 (39%)	16 (26%)	34 (52%)	
Worked independently with no or very little supervision	28 (22%)	8 (13%)	20 (30%)	
Meeting a clinical teacher from the university, *n* (%)				
Yes	91 (71%)	38 (61%)	53 (80%)	.020^b^
No	37 (29%)	24 (39%)	13 (20%)	
Satisfaction with clinical practice, md (q_1_, q_3_)	4 (4, 5)	4 (3, 5)	4, 5 (4, 5)	.27^c^

^a^ Number of weeks with full‐time studies.

^b^ Chi‐squared test.

^c^ Mann–Whitney U‐test.

### 4.3 The clinical learning environment

The district nursing students' evaluations of the clinical learning environment in their clinical placement were generally positive (Table 2). However, the statistical analyses showed that the pedagogical atmosphere was perceived statistically significantly less positively by those students who had performed their clinical placement in community‐based home care compared to those who had performed their clinical placement in primary health care. The students also differed statistically significantly with respect to their ratings of statements concerning sufficiently meaningful learning situations, the multidimensional content of the learning situations, the extent to which the learning atmosphere was positive, and whether their clinical placement could be regarded as a good learning environment. There were also statistical differences between the students concerning their evaluations of the leadership style of the ward manager. In particular, students practicing in community‐based home care agreed less than the other students with the statement that the manager was a team member and that his or her feedback could be regarded as a learning situation. The students also differed in their ratings of the premises of nursing in their clinical placement. District nursing students who had clinical placements in community‐based home care agreed less with statements that the documentation was clear and that information related to patients' flow of care was unproblematic than students who had clinical placements in primary health care. There were no statistical differences between the two groups of district nursing students about their evaluations of the supervisory relationship and the role of the nurse teacher from the university (Table 2).

**TABLE 2:** Differences between student nurses' assessment of the clinical learning environment in community‐based home care (*n* = 66) and primary healthcare centres (*n* = 62)

	Total (*n* = 128), m (*SD*)	Community‐based Home care (*n* = 62), m (*SD*)	Primary healthcare centres (*n* = 66), m (*SD*)	*p*‐value
Pedagogical atmosphere	4.0 (0.7)	3.8 (0.7)	4.1 (0.7)	.005
The staff were easy to approach	4.4 (0.8)	4.4 (0.8)	4.5 (0.8)	.246
I felt comfortable going to the ward at the start of my shift	4.3 (0.9)	4.2 (1.0)	4.5 (0.9)	.071
During staff meetings (e.g. before shifts) I felt comfortable taking part in the discussion	3.7 (1.1)	3.8 (1.0)	3.5 (1.1)	.089
There was a positive atmosphere on the ward	4.0 (1.0)	3.9 (1.0)	4.2 (0.9)	.034
The staff were generally interested in student supervision	3.7 (1.0)	3,6 (1.0)	3.8 (1.0)	.252
The staff learned to know the students by their personal names	4.1 (1.0)	4,1 (1.0)	4.1 (1.1)	.834
There were sufficiently meaningful learning situations on the ward	3.7 (1.1)	3,1 (1.0)	4.2 (0.9)	<.001
The learning situations were multidimensional in terms of content	3.7 (1.0)	3,3 (1.0)	4.0 (0.9)	<.001
The ward can be regarded as a good learning environment	3.9 (1.0)	3,7 (1.0)	4.2 (0.9)	.005
Leadership style of the ward manager (WM)	3.4 (0.9)	3.1 (0.9)	3.6 (0.9)	.001
The WM regarded the staff on her/his ward as a key resource	3.9 (1.0)	3.7 (1.0)	4.1 (1.0)	.017
The WM was a team member	3.1 (1.2)	2.7 (1.2)	3.6 (1.1)	<.001
Feedback from the WM could easily be regarded as a learning situation	3.0 (1.1)	2.7 (1.0)	3.2 (1.1)	.020
The effort of individual employees was appreciated	3.5 (1.0)	3.3 (1.0)	3.7 (1.0)	.029
Premises of nursing	3.6 (0.7)	3.5 (0.8)	3.8 (0.7)	.011
The ward's nursing philosophy was clearly defined	3.1 (1.0)	3.0 (1.1)	3.2 (1.0)	.236
Patients received individual nursing care	4.3 (0.7)	4.2 (0.7)	4.3 (0.8)	.293
There were no problems in the information flow related to patients' care	3.5 (1.0)	3.2 (1.1)	3.7 (0.9)	.015
Documentation of nursing (e.g. nursing plans, daily recording of nursing procedures, etc.) was clear	3.7 (1.0)	3.4 (1.1)	4.0 (0.9)	.006
Supervisory relationship	4.2 (0.8)	4.2 (0.9)	4.3 (0.8)	.656
My supervisor showed a positive attitude towards supervision	4.3 (0.9)	4.2 (1.0)	4.3 (0.9)	.218
I felt that I received individual supervision	4.1 (1.0)	4.1 (1.1)	4.2 (1.0)	.631
I continuously received feedback from my supervisor	3.9 (1.1)	3.9 (1.2)	3.9 (1.1)	.854
Overall I am satisfied with the supervision I received	4.1 (1.1)	4.0 (1.2)	4.2 (1.0)	.466
The supervision was based on a relationship of equality and promoted my learning	4.3 (0.9)	4.3 (0.9)	4.3 (0.9)	.600
There was a mutual interaction in the supervisory relationship	4.3 (0.9)	4.3 (0.9)	4.4 (0.9)	.371
Mutual respect and approval prevailed in the supervisory relationship	4.4 (0.9)	4.4 (0.9)	4.5 (0.8)	.348
The supervisory relationship was characterized by a sense of trust	4.4 (0.9)	4.4 (0.8)	4.4 (0.9)	.472
Role of the nurse teacher (NT)^a^	3.3 (0.9)	3.3 (0.9)	3.3 (0.8)	.775
In my opinion, the NT was capable of integrating theoretical knowledge and the everyday practice of nursing	3.6 (1.0)	3.6 (0.9)	3.6 (1.1)	.879
The NT was capable of operationalizing the learning goals of this clinical placement	3.7 (1.1)	3.7 (1.0)	3.6 (1.1)	.405
The NT helped me to reduce the theory‐practice gap	3.3 (1.1)	3.2 (1.1)	3.4 (1.1)	.500
The NT was like a member of the nursing team	2.3 (1.4)	2.5 (1.6)	2.2 (1.3)	.482
The NT was capable of giving his or her pedagogical expertize to the clinical team	2.8 (1.3)	2.8 (1.3)	2.8 (1.3)	.983
The NT and the clinical team worked together to support my learning	2.8 (1.4)	2.9 (1.4)	2.8 (1.4)	.695
The common meetings between myself, my mentor and the NT were a comfortable experience	4.1 (0.9)	4.0 (0.9)	4.1 (0.9)	.375
The climate of the meetings was congenial	3.6 (1.2)	3.6 (1.2)	3.7 (1,1)	.845
The focus of the meetings was on my learning needs	3.8 (1.0)	3.7 (1.0)	3.9 (0.9)	.143

^**a**^ Mann–Whitney U‐test.

### 4.4 Qualitative results

**FIGURE 1:**
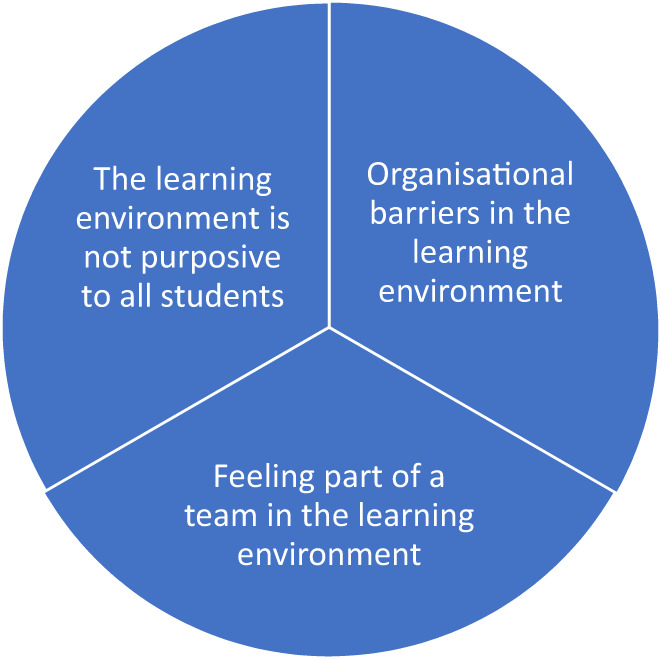
Three themes of the qualitative data.

The open‐ended questions concerning facilitators of and barriers to learning in the clinical environment were answered by 94 district nursing students, mainly with brief comments, of which half were related to the questions. The analysis revealed three themes: the learning environment is not purposive to all students, organizational barriers in the learning environment, and feeling part of a team in the learning environment, see Figure 1.

### 4.5 The learning environment is not purposive to all students

The district nursing students considered it problematic that they had to complete the same placements as students who already had work experience in primary health care or community‐based home care. They also claimed that their placement was not purposive since it did not provide them with new knowledge and perspectives. In addition, they desired for their work experience to be more fully considered. As one district nursing student expressed:

We who have already worked and have experience with primary healthcare centres can be given other tasks or courses instead. Now I have to be on site to get a grade, and I can't develop in another area, which I would prefer. (UO16)

### 4.6 Organizational barriers in the learning environment

The district nursing students claimed that the working climate at their clinical placement affected their opportunities to learn. Such organizational barriers were mainly related to a working environment that imposed psychosocial strain and high levels of stress on the staff. A stressful working environment can result in a disorderly workplace and staff dissatisfaction, both of which can negatively affect the students. Other barriers to an effective learning environment were supervisors and other staff being occupied by meetings and training to use new unfamiliar systems for care documentation and other IT‐related tasks. Staff shortages were also an issue, as one student explained:

Staff shortage made things messy and stressful. (VV09)

### 4.7 Feeling part of a team in the learning environment

The students articulated that one facilitator of a good learning environment was feeling welcomed by staff at their clinical placement. They felt that it was personally beneficial to be engaged by preceptors and other staff in workplace activities and to feel like a team member, as one student expressed:

To feel welcome and as one of the crew, so you could focus more on learning. (HB02266)

## 5 DISCUSSION

The district nursing students in this study were generally satisfied with the clinical learning environment in their clinical placement. That said, statistically significant differences were found in their experience of sufficiently meaningful learning situations and the multidimensional content of learning situations, with students in primary health care being more satisfied than students in community‐based home care. Meaningful learning situations entail the incorporation of pre‐existing knowledge and learning contexts (Cadorin et al., 2014). Our study revealed that all district nursing students had to complete a clinical placement in both primary health care and community‐based home care, even when they already had work experience as nurses in these clinical settings. The students also said that their placement was not always purposive since it did not provide them with new knowledge and perspectives. Backes et al. (2018) argued that preceptors must problematize their practice and base it on new questions to support the development of students' practice skills. By promoting reflection and critical thinking, preceptors can also help students identify interconnections and intersections between new and other forms of knowledge. In one study, the successful completion of a master's degree was found to have a positive effect on nurses' personal and professional confidence, cognitive functioning and development of evidence‐based practice (Watkins, 2011). This indicates that district nursing students' preceptors should have a specialist nurse degree and a master's degree in order to be sufficiently competent to ask questions that support students in problematizing practice experiences.

Our study also showed that a positive atmosphere was related to a good learning environment as it enhanced district nursing students' opportunities to learn during their clinical placement. Facilitators of a good learning environment included feeling like a team member and feeling welcomed at a clinical placement. Similar findings have been reported elsewhere (Rodríguez‐García et al., 2021; Sundler et al., 2014).

Barriers related to a bad working environment included the imposition of psychosocial strain and high levels of stress on the staff, as it can impede district nursing students' learning. Jarnulf et al. (2019) described precepting in two ways: conveying knowledge to students, and allowing students to reflect on their learning, including giving students time to listen to and have discussions with other students, and pursuing a reflective approach, one that stimulates critical thinking. There is a risk that the first way of precepting is dominant when the staff is stressed, as the other way of precepting demands time and engagement in the students' learning. To prepare students for their future work as district nurses in an evolving community, Ginger and Ritchie (2017) recommended that curricula and learning plans be used to support preceptors to ensure quality learning experiences. Preceptors for postgraduate students should be aware of the high demands in learning plans concerning knowledge, skills and capacity (Jarnulf et al., 2019). Furthermore, learning plans also demand that the organization for which the preceptor works provides sufficient time for supervision.

It is notable that district nursing students who performed their clinical placement in community‐based home care were statistically significantly less satisfied compared to students in primary health care. It was more common for students in primary health care to have worked independently and to have met with a nurse teacher from the university during their clinical practice.

Our results are in line with those of Nyhagen and Strøm (2016), who reported that postgraduate students prefer preceptors who challenge them to take initiative and work independently with patients. From a nursing students' perspective, Löfmark et al. (2012) reported that positive and beneficial supervision from both preceptors and teachers contributed to a large extent to the fulfilment of intended learning outcomes. Other research has shown that nurse teachers from university mostly support students and preceptors in evaluation situations through opportunities to talk to teachers directly (Hallin & Danielson, 2009). In light of the COVID‐19 pandemic, it is relevant to ask whether nurse teachers from universities can support both preceptors and district nursing students via digital technology instead of face‐to‐face meetings. Digital technology that allows participants to see and speak with each other and to share screens to, for example assignment documents. Although digital technology cannot replace face‐to‐face learning, it still permits mentoring by nurse teachers (Heinonen et al., 2019) and opportunities for networking and sharing for preceptors (Zournazis & Marlow, 2015). Our findings indicated that it was important for district nursing students and preceptors to meet with a nurse teacher from university to receive support and thereby strengthen preceptors in their role and students in their learning in clinical practice. Such meetings can promote sufficiently meaningful and multidimensional learning situations in clinical placements for postgraduate students.

### 5.1 Study limitations

There were limitations to this study. Even though the five universities followed national regulations, there were minor differences about the organization of postgraduate district nurse education. Although the location of clinical placements in primary health care and community‐based home care could be standardized, the length of clinical practice could not. The qualitative open‐ended questions yielded a preliminary understanding of the phenomenon, which can be expanded further in future studies.

## 6 CONCLUSIONS

Our study showed that postgraduate district nursing students value the clinical learning environment in primary health care more than that in community‐based home care. The findings indicated that students who had previous work experience as nurses had greater demands concerning what constituted meaningful learning situations. To further develop their learning from clinical practices, these students need preceptors who have the skills and competence required to support more advanced reflections and critical thinking in caring situations. To create such situations, preceptors should consider and clarify the multidimensional content in learning situations in order to create a more positive clinical learning environment. It seems like such learning can be facilitated by preceptors who had a specialist nurse degree at the master's level rather than qualifications lower than those of the students. Multidimensional content in learning situations benefit district students learning and a positive atmosphere is useful for creating a good learning environment.

## AUTHOR CONTRIBUTIONS

ML, AS, KB, BB involved in conceptualization and methodology. AS and BB involved in formal analysis, software, validation and visualization. ML and BB wrote the original draft. ML, AS, BB and KB involved in reviewing and editing.

All authors have agreed on the final version and meet at least one of the following criteria [recommended by the ICMJE (http://www.icmje.org/recommendations/)]:

- substantial contributions to conception and design, acquisition of data or analysis and interpretation of data;
- drafting the article or revising it critically for important intellectual content.

## FUNDING INFORMATION

No external funding. The respective universities funded their scientists.

## CONFLICT OF INTEREST

None.

## Data Availability

The data that support the findings of this study are available on request from the corresponding author. The data are not publicly available due to privacy or ethical restrictions.
